# An Acute Respiratory Infection of a Physiologically Anemic Infant is a More Likely Cause of SIDS than Neurological Prematurity

**DOI:** 10.3389/fneur.2016.00129

**Published:** 2016-08-23

**Authors:** David T. Mage, Maria Luisa Latorre, Alejandro G. Jenik, E. Maria Donner

**Affiliations:** ^1^Retired, Newark, DE, USA (formerly affiliated to WHO, Geneva, Switzerland); ^2^Corporacion Infancia Colombia, Bogotá, Colombia; ^3^Hospital Italiano, Buenos Aires, Argentina; ^4^Dupont Haskell Laboratory, Newark, DE, USA

**Keywords:** SIDS, X-linkage, 4-parameter lognormal distribution, live-birth order, acute respiratory infections, physiologcal anemia

## Abstract

**Introduction:**

The cause of the sudden infant death syndrome (SIDS) is perhaps the oldest of unsolved mysteries of medicine, possibly dating back to Exodus in Biblical times when Egyptian children died in their sleep as if from a plague. It occurs when infants die unexpectedly with no sufficient cause of death found in a forensic autopsy, including death scene investigation and review of medical history. That SIDS is an X-linked recessive death from infectious respiratory disease of a physiologically anemic infant and not a simple anomalous cardiac or neurological condition is an extraordinary claim that requires extraordinary evidence. If it were by a simple cause, it would have already been solved, with over 11,000 papers on SIDS listed now in PubMed. Our aim is to use mathematical models of SIDS to explain: (1) its 50% excess male death rate; (2) its 4-parameter lognormal distribution of ages at death; (3) its winter maxima and summer minima; and (4) its increasing rate with live-birth order.

**Methods:**

From extensive SIDS vital statistics data and published epidemiologic studies, we developed probability models to explain the mathematical behavior of SIDS meeting the four constraints mentioned above. We, then, compare these SIDS properties to infant death from acute respiratory infection (ARI), and infant death from encephalopathy, unspecified (EU).

**Results:**

Comparisons show that SIDS are congruent with ARI and are not consistent with EU and that these probability models not only fit the SIDS data but they also predict and fit the male fraction of *all* infant and child mortality from birth through the first 5 years of their life.

**Conclusion:**

SIDS are not rejected as an X-linked disease involving ARI and are not explained by a triple risk model that has been commonly accepted by the SIDS medical community, as implicating a neurological causation process in a subset of SIDS.

## Background

The sudden unexpected death of an apparently healthy and well-nurtured infant or young child during sleep – that in modern times remains unexplained after forensic autopsy, medical history review, and death scene investigation – is a phenomenon that has appeared throughout human history. It only became known as the sudden infant death syndrome (SIDS) in 1969. In Exodus (11:4–6), the Bible records a plague of such deaths in Egypt that were given a religious supernatural explanation. The history of SIDS is replete with hundreds of theories for its explanation. They range from overlaying of the infant by the mother falling asleep while nursing, suffocation from head covering, thymic asthma from a large thymus occluding the trachea, cardiac failure from long QT syndrome, to neurological deficits of the serotonergic system of the brain stem (1). Until now, even with new and advanced diagnostics, modern medical science has still been unable to discern the SIDS cause or discover a common identifying internal factor other than the defining absence of any apparent and sufficient cause of death. The aim of our paper is to show through mathematical modeling that an occult acute respiratory infection (ARI) plays a major role in the succession of events that lead a child to die suddenly and unexpectedly, without any explanation.

“Any viable hypothesis for the cause of SIDS must account for its characteristic age distribution” (2). A left-censored 4-parameter lognormal (a.k.a. Johnson S_B_) age distribution fits these age data and predicts that SIDS is negligible at birth, rises to a maximum rate between 2 and 3 months of completed life, and goes to 0 at or about 3.5 years (3). Note that the limitation of SIDS to ages under 1 year in recent years is a ‘legal fiction’ for research purposes only (4). We reason that SIDS must involve age-varying risk factors that are necessary but insufficient-alone to cause SIDS, including some that are not measured at autopsy, and, collectively, they create that age distribution.

For example, physiological anemia is a natural phenomenon that occurs when fetal hemoglobin (HbF) disappears faster than it is replaced by adult hemoglobin (HbA). Hemoglobin (Hb) is not measured at autopsy because of hemostatic gravitational settling of red blood cells leading to lividity and also because it is a natural phenomenon that is compensated for by infants increasing heart rate to maintain oxygen throughput to the brain (5, 6). For term infants at birth, the mean Hb is about 16.5 g/dl with a SD σ of about 2 g/dl. The mean Hb falls to its nadir of 10.5 g/dl with σ = 1.5 g/dl at or about 8 weeks, the age at which SIDS has its peak rate. However, there is no margin of safety for those 2% of the infants with the lowest total Hb (<−2σ). We note the high Hb at birth can explain the absence of SIDS in the first days of life when most other causes of infant death from neurological immaturity have their highest rates.

Whereas traditional medicine repeatedly autopsies SIDS over and over again, expecting to find its cause, we took an engineering approach and looked to the numerical structure of SIDS vital statistics data for an insight. We propose that probability models of risk factors show that: (1) an X-linkage may create the 50% male excess SIDS rate (7, 8); (2) the observed same 4-parameter lognormal age distribution for both males-and-females and prone-and-supine sleepers, is predicted by Cramér’s Theorem (3, 9, 10) (NB: because total of all SIDS ages have a normal transform distribution, any subsets of SIDS must also have the same normal transform distribution); (3) SIDS and ARI have the same seasonal pattern (11); and (4) the increasing SIDS rate with live-birth order (LBO) is related to increased probability of ARI brought home to the infant by family members (12, 13). The X-linkage model can then predict the 5/9 male fraction of *all* infant mortality for equal numbers of males and females at risk (14–16).

Given that an infant put to bed to sleep is found dead in exactly the same circumstances as for the immediately preceding sleep period that was survived, one has to ask, “*why was that night different from any other night*” to cause the infant to die in just that interval? The SIDS’ parents or other caregivers have no premonition that their infant is at immediate risk of imminent death, so the precipitating fatal event in SIDS must occur suddenly without warning, or they would have sought prompt medical help. We propose it is an occult prodromal respiratory infection that fulminates lethally in the infant (12) with unmeasured asymptomatic physiological anemia (total Hb < −2σ), autopsied without blood or lung viral or bacterial cultures (17), or with such testing leading to a culture-negative sepsis (12, 18). “Apart from the problems resulting from post mortem effects, culture, immunofluorescence, and ELISA tests are known to give significant false negative (FN) rates” (19). An hypothesized recessive *q* = 2/3 X-linkage could allow acute anoxic encephalopathy, perhaps with apnea, to occur in possibly immature neurons, or a deficient number of respiratory control neurons of the brain stem, and the infant never awakens. If the complimentary dominant X-linked allele with frequency *p* = 1 − *q* = 1/3 could provide for an enzyme that would allow the respiratory control neurons to switch from aerobic to anaerobic oxidation, the infant could survive the transient anoxia. A recent study to identify this possibility could not identify such a gene locus involved, perhaps because the Illumina platform used only covered an estimated 90–95% of the X-chromosome (20, 21). We explain below why autosomal–androgen interactions are unlikely to play a role (14).

The current SIDS literature, as exemplified by papers in this very Frontiers topic, still considers a published “Triple Risk Model” (TRM) as possible for a subset of SIDS (22), even though, there is no known, common internal marker of susceptibility, no common external factor of risk, and no common restriction of SIDS ages to a distinct sub-period of SIDS susceptibility – because a single equation covers the entire age range from birth to 3.5 years (3). Such a TRM with congenital neurologic immaturity and underdevelopment of the serotonergic neurological systems would have maximal danger at or immediately after the birth as do other congenital anomalies, such as encephalopathy, unspecified (EU), whereas SIDS has a minimal rate there (23). Conversely, our proposed model with the effects of maternal iron-deficiency anemia *in utero*, delaying neurological development (24, 25), and leading to severe physiological anemia *ex utero* (5, 6) has the anemia, not the neurological deficits, playing a causative role. This anemia has the infants presenting with their maximal Hb at birth that may explain this unique property of minimal neonatal SIDS and the neurological underdevelopment observed in a subset of SIDS (22). That is, the same maternal iron-deficiency anemia may cause both developmental delays in the infant’s monoaminergic systems [including serotonin (5-HT) transporters] and the infant’s relatively low postnatal Hb – leading to a fatal cerebral anoxia, so their correlation may be mistaken for the causation of SIDS.

We, now, discuss the four factors cited above (gender, age, seasonality, infectivity) that can explain SIDS and, then, we predict the total male fraction of *all* infant mortality that support our probability models for the cause of SIDS.

### Gender and the 50% Male Excess of SIDS

As stated by Naeye et al. (7), “The general disadvantage of male infants has long been recognized. The biologic difference *must* originate in the genetic differences between the sexes and those genetic differences are the consequence of disparity in the number of the X chromosomes … This gives the female options for variability not open to the male.” Table 1 shows the male fraction of SIDS and other respiratory infant deaths and diseases that all seem to fluctuate about a value of 0.612 for the male fraction of SIDS. We know of no mechanism other than a recessive X-linkage in Hardy–Weinberg equilibrium (HWE) that can cause such a constant excessive male fraction of infant mortality. Whereas there may be autosomal–androgen interactions that can lead to a male excess for conditions, such as cleft lip and male pattern baldness, we have shown that the same 50% male excess occurs monthly throughout the first year of life, while testosterone rises and falls in the months after birth to aid in the descent of the testes into the scrotum (14).

**Table 1:** **Male fractions of SIDS and other respiratory diseases showing the same infant male fractions of order 0.61 (26)**.

Authors	Diseases	Male mortality	Female mortality	Male fraction
CDC (27); U.S.; 1968–2014	Suffocation by inhalation of food or foreign object <5 years	8,940	6,070	0.596
Carpenter and Gardner (28); England and Wales; 1965–1976	Sudden respiratory death (70% SIDS) – at home	11,212	7,443	0.601
Carpenter and Gardner (28); England and Wales; 1965–1976	Respiratory deaths not sudden – in hospital	2,375	1,564	0.603
Naeye et al. (7); U.S.	Total neonatal <72 h (less antenatal aspiration identified by squamous cells in terminal airspaces)	1,009	660	0.604
Fard et al. (29); Hannover	SIDS	163	104	0.610
Mage and Donner (8); Global	SIDS (36 data sets)	41,238	26,140	0.612
Carpenter et al. (30); Europe, NZ	SIDS	1,466	898	0.613
Gupta et al. (31); Scotland; 1982–1990	Bronchiolitis: hospital discharge diagnoses	6,127	3,881	0.614
Wilkinson and Skuza (32); Australia; 1981–2000	SIDS	4,402	2,752	0.615
CDC (27); U.S.; 1968–2014	Respiratory distress syndrome	98,328	61,790	0.619
Gupta et al. (31); Scotland; 1982–1990	SIDS	751	460	0.620
Total	All the above	176,011	110,962	0.613

It is interesting to note that Guntheroth (1) in a table of the most important epidemiologic facts on SIDS did not include the constant male fraction of 0.61, as shown in Table 1 (33). We proposed (8) that this male fraction of 0.61 could be caused by a non-protective X-linked recessive allele with frequency *q* = 2/3 and a protective dominant corresponding X-linked allele with frequency *p* = 1/3. The XY male would be at risk with frequency *q* = 2/3 and the XX female would be at risk with frequency *q*^2^ = 4/9, giving the male a 50% excess risk for equal numbers of males and females at risk (3 males:2 females). However, there is a male live-birth excess rate of order 5% that has a slight variation from country to country and from year to year within a country, so that there would be 3.15 males:2 females, giving a male fraction of order 3.15/(3.15 + 2) = 0.6117. Therefore, some of the variance in the male fractions of Table 1 may be due to those fluctuations at or about the nominal male live-birth excess of 5%. We also noted that Carpenter and Gardner (28) reported in their Table 1, for England and Wales 1965–1976, that all infant deaths from non-respiratory anomalies were 5,653 males and 5,369 females, a 5.3% male excess, similar to the nominal 5% male live-birth excess. This should have indicated to those looking for autosomal variants related to SIDS that androgen interactions were also unlikely.

### The 4-Parameter Lognormal Distribution of the SIDS Ages

**Figure 1:**
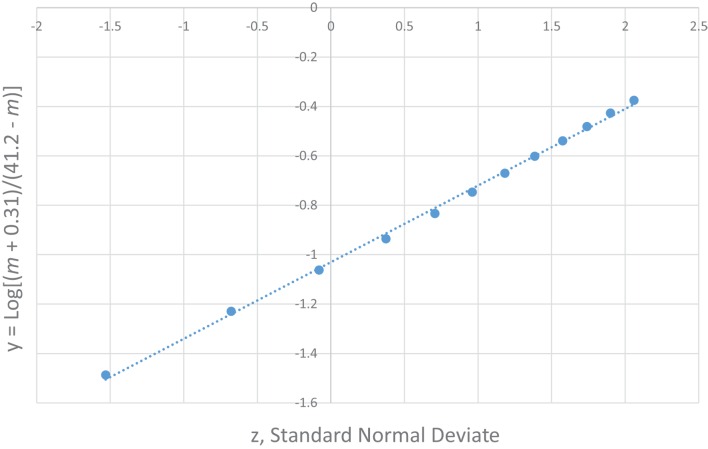
**4-parameter lognormal distribution of SIDS ages (34)**. *y* = 0.31 *z* − 1.03.

The most unique characteristic of SIDS is its 4-parameter lognormal age distribution (a.k.a. Johnson S_B_) that must be explained by any theory for the cause of SIDS (2, 3). The equation we developed for SIDS is as follows: *y* = Log [(*m* − [−0.31])/(41.2 − *m*)] = μ + σ *z*, where *m* is age in months, μ is the value of *y* at the median point, σ is the SD of *y*, and *z* is a standard normal deviate (Note, the negative third parameter −0.31 requires the distribution to be censored at *m* = 0). Table 2 shows the age distribution data for multiple SIDS studies combined (34) and Figure 1 is the frequency distribution of *y* vs. *z* described by a 4-parameter lognormal distribution (*y* = 0.31 *z* −1.03).

**Table 2:** **The age distribution of SIDS – multiple studies pooled together (34)**.

Age *m*^a^	1	2	3	4	5	6	7	8	9	10	11	12	13–41	*N*
*y*	−1.487	−1.230	−1.060	−0.936	−0.834	−0.747	−0.670	−0.602	−0.539	−0.481	−0.427	−0.375		
*n*	3,550	10,496	12,354	10,036	6,436	4,046	2,811	2,007	1,430	940	683	511	1,110^b^	56,410

*^a^Months are defined differently by different authors as 1,461 days/48 months is not an integer*.

*^b^Estimated by semi-logarithmic extrapolation of numbers at 4 – 12 to 41 months*.

**Figure 2:**
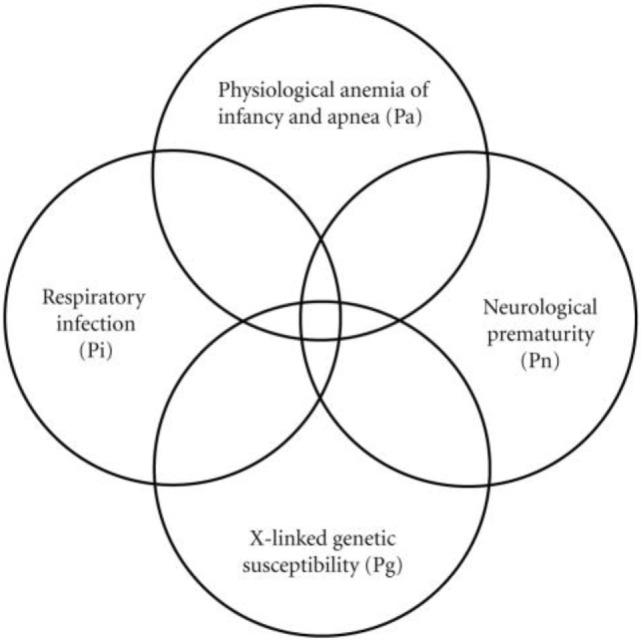
**Venn diagram explaining the generation of the 4-parameter lognormal age distribution of SIDS satisfying Cramér’s Theorem (3, 9, 10, 35)**.

To explain the generation of this observed 4-parameter lognormal distribution as required for any correct explanation of SIDS (2), we developed a Venn diagram (Figure 2) for a quadruple risk model of SIDS (35). The four probability factors involved with SIDS discussed in this paper explain the age and gender distributions invariant with different sleep position and subsets of SIDS found with and without neurological prematurity (Pn) and respiratory infection (Pi). It is proposed that a prone infant is susceptible to SIDS anywhere in the intersection between the genetic (Pg) and anemia-related apnea (Pa) factors with *either* Pn *or* Pi, but a supine sleeping infant is only susceptible to SIDS if it is in the intersection of all four factors (Pa, Pg, Pi, *and* Pn). This is easily explained mathematically from our model as follows: let there be two causal-risk factors, one with probability increasing with age in months (*m*) as Pi = 0.31/(41.2 − *m*) and the other with probability decreasing with age as Pn = 0.31/(*m* + 0.31). For supine sleep, let the infant require both simultaneously with the probability equal to their product as PiPn = 0.1/[(41.2 − *m*)(*m* + 0.31)]. For prone sleep, let the infant only require one of them, which will have the probability approximately equal to their sum Pi + Pn, as 0.31[1/(41.2 − *m*) + 1/(*m* + 0.31)]. However, this sum can be rewritten as equal to 0.31 [(41.2 − *m*) + (*m* + 0.31)]/[(41.2 − *m*) (*m* + 0.31)] = 12.9/[(41.2 − *m*)(*m* + 0.31)] that has the same form as for supine sleep, varying only by the constants 0.1 and 12.9.

### Seasonal Pattern of SIDS

**Figure 3:**
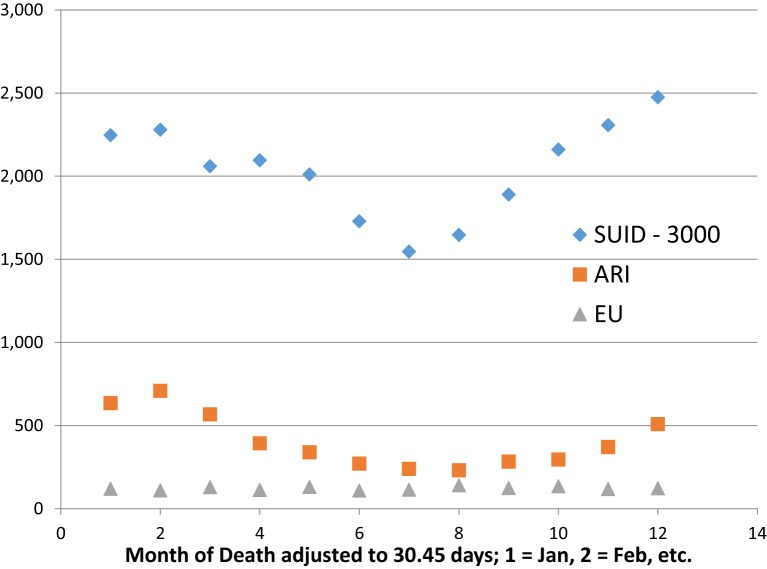
**Seasonal variation of SUID and ARI but not EU**. U.S. 1999–2014, http://wonder.cdc.gov

Sudden infant death syndrome has a known seasonal pattern, with maximal rate during the cold winter and minimal rate during the warm summer months found in Europe and North America. CDC (27) reports the monthly numbers of SIDS, ARI, and EU for 1999–2014. We sum the numbers reported by calendar month (28–31 days) and adjust these totals to a fixed month length of 1,461 days/48 months = 30.44 days, by multiplying each total by 30.44/days per month. Table 3 shows the ICD-10 numbers for sudden unexpected infant deaths {SUID = [SIDS (R95), UNK (R99), accidental suffocation strangulation in bed (ASSB) (W75)]}, ARI (J00-J26), and EU (G93.4). Figure 3 shows that SUID and ARI have similar sinusoidal variation, but EU does not.

**Table 3:** **Seasonal variation of SUID, ARI, and EU, U.S. 1999–2014 (27)**.

Disease	January	February	March	April	May	June	July	August	September	October	November	December
SUID	5,247	5,280	5,061	5,096	5,012	4,730	4,547	4,647	4,891	5,161	5,308	5,476
ARI	636	710	568	393	340	272	240	231	284	296	372	509
EU	121	111	130	114	131	109	115	141	125	135	119	123

Seasonality in SUID/SIDS is not a function of ambient temperature variation between winter and summer. This was shown by Mage (11) looking at semi-tropical Hawaii, which has negligible temperature variation throughout the year. However, it does have seasonal variation of SIDS that was attributed to the tourist influx during the year from the U.S. and Japan where there is ARI seasonal fluctuation that is transmitted to the Hawaiian population. The South American country Colombia is also semi-tropical with wide changes in elevation, but no cold winter. There, the respiratory infection peak occurs during the rainy season that matches their SUID peak (36).

### Respiratory Infection and SIDS

Infants are normally and routinely placed to sleep by their parents in a habitual customary manner. In the case of SIDS, the parents or other caregivers have no premonition that the infant is at imminent risk of dying. The baby’s clothing, sleep position, bed and bedding, and other items, such as pacifier use, room temperature, and feeding pattern will be very like the infant’s normal pattern in the immediately previous sleep period. One needs to ask “*what then could have changed between the immediately previous sleeping conditions and the final sleep from which the infant never awoke?*” We propose that virtually the only likely thing that could have changed, which is capable of causing a sudden death, is the rapid fulmination of an occult prodromal respiratory infection that was invisible to the person placing the infant to sleep (12).

Infants who catch a respiratory infection must get it from contact with a carrier of that communicable infection, which is most likely a cohabiting family member (CFM) (37). For infants of a given LBO, we assume that they live with two parents and all LBO – one older siblings, so that CFM = LBO + 1. Table 4 shows combined global data from four studies from the U.S. (28, 38), Europe (30), and Colombia (39) of SUID. SUID was defined by CDC (27) for ICD-10 as R-95 SIDS, R-99 Unknown cause or SIDS with an incomplete forensic investigation (UNK) and W-75 ASSB – with possible suffocation from prone sleep position. As readily seen from the next to last column, the rate of total SUID increases, monotonically, with LBO and our estimate of CFM.

**Table 4:** **Four combined studies of global SUID = SIDS + UNK + ASSB (13)**.

Live-birth order (CFM)	SIDS + UNK + ASSB	Infants at risk	SUID rate per 1,000	SUID model per 1,000 = 3.60*(1–0.9CFM)
0 (0)	0	0	0	0
1 (2)	27,945	38,759,660	0.7210	0.6843
2 (3)	30,037	30,465,292	0.9859	0.9760
3 (4)	18,288	15,418,084	1.1861	1.2386
4 (5)	9,539	6,467,509	^a^1.4749	^a^1.4749
5 (6)	3,327	1,989,679	1.6721	1.6876
6 (7)	2,818	1,520,300	1.8536	1.8790

*^a^Model fit by matching to this datum point*.

**Figure 4:**
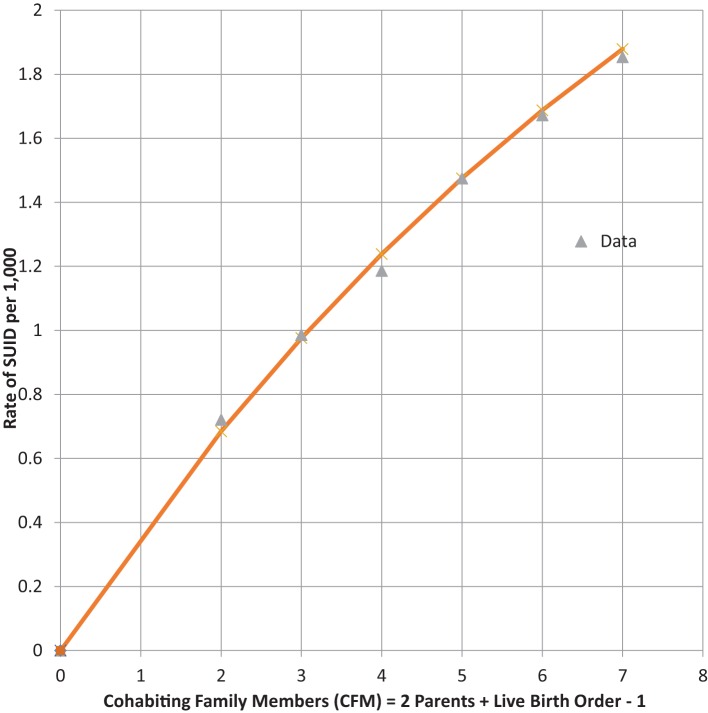
**SUID rate per 1,000 increasing with family size**. (CFM) Global Rate = 3.60*(1 − 0.9^CFM^).

We noted the increase of SUID rate as LBO/CFM increases and have developed the following probability model to express the concave shape of the relationship: let *P* equal the average probability of a family member *not* being a carrier of a communicable respiratory infection, the probability that all CFM are non-infective will then be equal to *P*^CFM^, and the probability that the infant will be exposed to *at least one* such CFM will be equal to 1 − *P*^CFM^. Our model fit to these data is that *P* = 0.9 and the SUID rate per 1,000 at risk with a given LBO, shown in the last column of Table 4, is as follows: rate per 1,000 = 3.60*(1 − 0.9^CFM^). Figure 4 shows the goodness of fit of this model to these data. Note how the model goes to the origin (0 SUID for a virtual cloned infant that has 0 CFM – as in Aldous Huxley’s *Brave New World*), smoothly without any discontinuity. Therefore, all SUID appear to be related to a possible source of an ARI.

**Figure 5:**
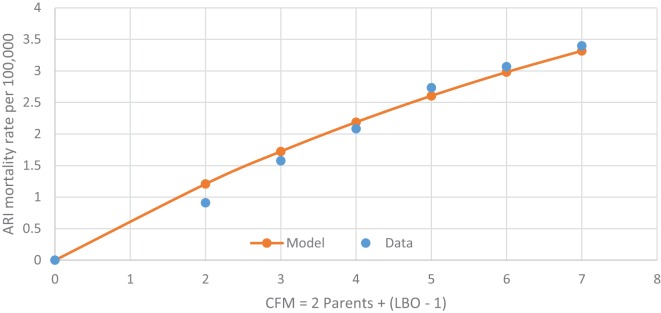
U.S. 1995–2013 ARI rate/100,000 increasing with CFM as a respiratory infection vector, with rate = 6.36*(1 − 0.9^CFM^).

Table 5 shows the U.S. infant mortality rate from both Upper and Lower ARIs from 1995–2013 (27). The corresponding codes are ICD-10, J00-J06, J20-J22, and ICD-9 460-466. As for SIDS, the ARI mortality rate increases with LBO in Figure 5 and with a similar mathematical relationship for prediction as from Figure 4. This supports our finding that SIDS appears to have a causal relationship to the initial fulmination of an occult prodromal ARI that may cause neuronal death in the physiologically susceptible infants (12). We note again that many U.S. medical examiners do not culture lung exudate of potential SIDS cases because “of a perceived lack of testing utility” (17), and that, in many cases, the cultures are negative in spite of other evidence of severe sepsis (18, 19). Indeed, Farber wrote, in some such cases, “no growth was obtained from the blood stream although gross and histological changes in these cases were identical with those in which a positive culture was found.” (12).

**Table 5:** **U.S. 1995–2013 (http://wonder.cdc.gov) acute upper and lower respiratory infection mortality (ICD-10, J00-J06, J20-J22, and ICD-9 460-466) (27)**.

Live-birth order (CFM)	ARI J00-J06, J20-J22 ICD-9 460-466	Infants at risk	Rate per 100,000	Model rate per 100,000 = 6.36*(1–0.9^CFM^)
0 (0)	0	0	0	0
1 (2)	280	30,740,193	0.9108	1.2084
2 (3)	387	24,528,771	1.5777	1.7235
3 (4)	265	12,707,378	2.0854	2.1872
4 (5)	139	5,081,597	2.7353	2.6044
5 (6)	58	1,890,067	3.0686	2.9800
6 (7)	50	1,470,973	3.3991	3.3180

In comparison to SIDS and ARI which have increasing risks of mortality with increasing family size as potential carriers of an ARI, infant mortality from neurological cell death in infants with underdeveloped brain structure at birth, from EU is independent of the infant’s family size or LBO. Table 6 shows U.S. EU data on this cause (27).

**Table 6:** **U.S. 1995–2013; brain cell death, encephalopathy, unspecified, in infants with neurological underdevelopment at birth (http://wonder.cdc.gov) ICD-10 G93.4, ICD-9 348.3 (27)**.

Live-birth order (CFM)	Encephalopathy, unspecified, ICD-10 G93.4, ICD-9 348.3	Infants at risk	Rate per 10,000	No appropriate model for EU
1 (2)	200	30,740,193	0.0651	–
2 (3)	152	24,528,771	0.0620	–
3 (4)	67	12,707,378	0.0527	–
4 (5)	29	5,081,597	0.0571	–
5 (6)	12	1,890,067	0.0631	–
6 (7)	13	1,470,973	0.0884	–

**Figure 6:**
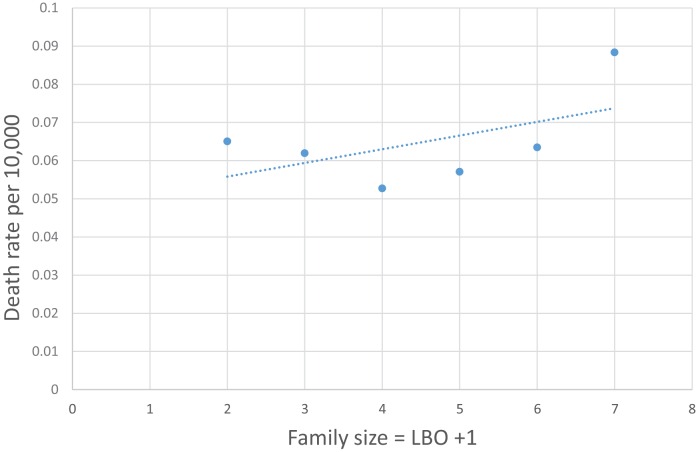
**U.S. 1995–2013 Encephalopathy, unspecified rate per 10,000 (27) not steadily increasing with family size (LBO + 1)**.

Figure 6 shows that the family size of the infant dying from neurological underdevelopment and immaturity at birth, with a diagnosis of EU, has no consistent relation to the rate of EU. In addition, whereas EU occurs predominantly (60%) in the first 4 weeks after birth, SIDS spares the neonate (3, 21). Thus, it is unlikely that neurological deficiencies from fetal underdevelopment of serotonergic brain cells in SIDS can be responsible for a large subset of SIDS. The reader should note that Cramér’s theorem requires that all subsets of SUID, such as SIDS, must have the same normal transform age distribution as SIDS that would also argue against a neurological subset (9).

**Figure 7:**
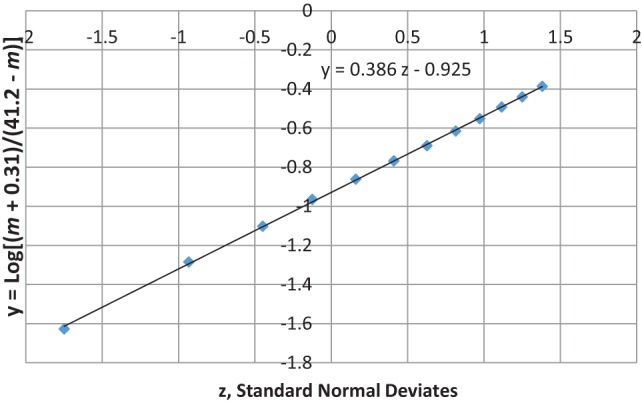
**Age distribution of bronchiolitis hospital admissions in Scotland, 1982–1990 (31)**.

**Figure 8:**
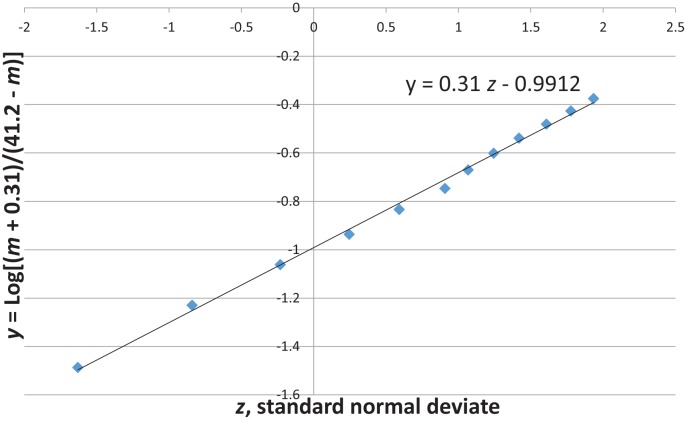
**SIDS in Scotland, 1982–1990 (31) *y* = Log[(*m* + 0.31)/(41.2 − m)] vs. *z*, standard normal deviate**.

Figures 7 and 8 show that the age distribution of hospital discharges for bronchiolitis and SIDS deaths, respectively, in Scotland 1982–1990, have the same lognormal form, as well as the same male fractions (SIDS 0.612 and bronchiolitis 0.614). Note that SIDS in Figure 8 have virtually the same slope and intercept as SIDS in Figure 1. Gupta et al. (31) came to the conclusion that “the two conditions do not appear to be closely related” by a chi-square test and analyses of their autocorrelation structures. They attributed the bronchiolitis hospitalization cases to the ubiquitous respiratory syncytial virus (RSV) to which virtually all infants are exposed by the end of the second year of life (37). However, there are several reasons why the statistical comparison between SIDS and RSV hospitalization may show no significant relation if one did exist:

All statistical testing assumes that measurements are made without error, but SIDS has false positives (FP) and FN. Thus, Recorded SIDS (RS) = Actual SIDS (AS) + False Positives (FP) − False Negatives (FN). In addition, not all ARI are caused by RSV (12, 37). Therefore, SIDS should have been compared by Gupta et al. to all infant hospitalization discharges for upper and lower ARI, not just those for bronchiolitis.

## Prediction of Male Fraction of *all* Total Infant Mortality from the X-Linkage Model for SIDS

All natural infant deaths occur either from cardiac failure (heart stops beating first) or respiratory failure (breathing stops first), neglecting trauma cases, such as fall from great height when both stop simultaneously. As described above, all infant mortality from respiratory causes has an apparent 50% X-linkage male excess rate for equal numbers of XY males and XX females at risk (we neglect chromosomal abnormalities, such as XXY male and XXX female, and assume no infanticides). As also shown, there is no male excess (0%) for cardiac failures, which include congenital anomalies related to genetic variants on the 22 autosomes, which males and females have with equal probabilities, assuming HWE (40, 41). That is, for every two females dying from respiratory causes, three males will die, and for every two females dying of cardiac causes, two males will die.

We, then, assumed that female infants are equally likely to die from cardiac failure as respiratory failure (14, 15). If so, then for every two females dying from respiratory failure there will be three males, and two males and two females dying from cardiac causes. Thus, for equal numbers of males and females at risk there will be five males dying for every four females dying from all natural causes, predicting a 5/9 = 0.55555 male fraction for *all* infant mortality. Given that there is a nominal 5% male excess live-birth rate, 5.25 males will die for every 4 female infants dying from all causes, predicting a male fraction of 5.25/9.25 = 21/37 = 0.567567. We assume that infants under 5 years neither play independently of adult supervision nor display the male hyperactivity that increases their death rates from accidents and trauma later as they age. We, therefore extend our analysis in Table 7 up to 5 years where data are available as in the U.S.

**Table 7:** **Total male and female infant deaths and male % excess at risk for the U.S. (27), 13 countries pooled together (16) Argentina (42), Colombia (39), Norway (43), and England and Wales (28)**.

Country	Years	Ages	Male excess, at risk, %	Total male mortality	Total female mortality	Male fraction
U.S.	1968–2014	<5 years	4.62	1,221,981	932,096	0.5673
13 countries	Various	<5 years	5.11	294,827	223,004	0.5694
England and Wales	1969–1976	<1 year	5.99	22,965	17,502	0.5675
Argentina	1980–2012	<1 year	5.28	263,680	203,802	0.5640
Colombia	1979–2012	<1 year	5.51	261,676	200,296	0.5664
Norway	1967–1988	<1 year	5.44	3,103	2,344	0.5697

*Predicted male fraction for 5% male excess at risk is 0.5676 and 0.5699 for 6%*.

We chose to report the male fractions without correcting for the male excess at risk fluctuations from 5% (16). For example, dividing total deaths by total births in the same periods would be misleading because, as for the U.S., some children below 5 years dying in 1968 would have been born in the previous 5 years and some born in 2014 would die in the next 5 years.

## Summary

The results shown above become visible when large sample sizes are created by pooling observations from different data sets. We propose that, because SIDS has no objective finding, the diagnoses are subjective, and different pathologists reviewing the same slides and findings will assign different causes of death (43). Consequently, FP and FN SIDS are likely not randomly created by a given pathologist so their case studies will have either a positive or negative bias. When independent SIDS data sets are pooled together, the FP and FN will tend to cancel out and, by the law of large numbers, the mean value of the errors will approach 0 as the total number of observations becomes large. In the pooled data sets, we have analyzed, the numbers of observations are very large totaling from tens of thousands up to a few million, so the means of the data sets closely approach the means of the underlying distributions.

As shown here, and in our other papers cited, the following mathematical relationships that SIDS display must be explained by any proposed cause for SIDS:

(1) The constant SIDS 50% male excess rate compared to the female rate for equal numbers of infants at risk. Given the nominal 5% excess male birth rate, this leads to the observed male fraction of 0.612 (8);
(2) The SIDS left-censored 4-parameter lognormal (Johnson S_B_) age distribution that has 3rd and 4th parameters of order −0.31 and 41.2 months, respectively, with median of approximately −1.0 and approximate SD of 0.30;
(3) Maximum SIDS rate in winter months and minimum rate in summer months (10);
(4) The increased risk of SIDS with the infant’s increasing numbers of older siblings, proportional to the factor (1 − 0.9^CFM^), where CFM = 2 parents + (LBO − 1) siblings (13, 14);
(5) The SIDS X-linkage model predicts the 5/9 male fraction of *all* infant mortality for equal numbers of males and females at risk (13, 14).

To the best of our knowledge, no other cause of SIDS has been proposed that meets these five essential conditions that are necessary, but insufficient, to prove that they are the cause of SIDS. For proof of its causation, the predicted missing X-linked *p* = 1/3 dominant allele that is protective of neuronal cell death by acute anoxic encephalopathy by enabling the infant to shift from aerobic to anaerobic oxidation must be identified (4, 14). This may be complicated because of the likely presence of FP SIDS in the study cohorts, where non-SIDS cardiac causes of death or cases of infanticide may have been missed. In addition, our model requires that the SIDS infant be in the lowest percentiles (<−2σ) of Hb from the natural physiological anemia that minimizes for all infants between 2 and 3 months of age (5, 6). However, due to the gravitational settling of the red blood cells during hemostasis leading to lividity, an accurate blood Hb cannot be measured. If all infants in a birth cohort had their blood Hb measured at birth, then, perhaps, the lowest Hb infants could be identified as the susceptible cohort and, if so, treated to increase their Hb (44).

## Conclusion

We propose that the most physiologic-anemic infants can be identified by measuring Hb at birth. Then, if an enzyme coded for by the putative protective dominant X-linked allele (that passes through the blood–brain barrier) can be identified and given to the unprotected infant, it may be possible to reduce infant mortality significantly, by reducing the numbers of infants dying from SIDS and all other respiratory causes (16).

## Author Contributions

DM prepared the first draft and developed probability models to fit the age and family data. ED provided the genetic model, discussed the first draft with DM and made revisions, and approved the final draft. AJ provided and discussed the vital statistics for Argentina, reviewed the first draft and commented on the medical aspects, and approved the final draft. ML provided and discussed the vital statistics for Colombia, reviewed the first draft, and commented on the medical aspects and approved the final draft.

## Conflict of Interest Statement

The authors declare that the research was conducted in the absence of any commercial or financial relationships that could be construed as a potential conflict of interest.

## Abbreviations

ARI, acute respiratory infection; ASSB, accidental suffocation strangulation in bed, CDC, Centers for Disease Control and Prevention; CFM, cohabiting family members; EU, encephalopathy, unspecified; HWE, Hardy–Weinberg equilibrium; Hb, hemoglobin; ICD, international classification of diseases; LBO, live-birth order; RSV, respiratory syncytial virus; SIDS, sudden infant death syndrome; SUID sudden unexpected infant death; TRM, triple risk model; UNK, unknown.
